# Heat stress during grain filling affects activities of enzymes involved in grain protein and starch synthesis in waxy maize

**DOI:** 10.1038/s41598-018-33644-z

**Published:** 2018-10-23

**Authors:** Huan Yang, Xiaotian Gu, Mengqiu Ding, Weiping Lu, Dalei Lu

**Affiliations:** Jiangsu Key Laboratory of Crop Genetics and Physiology/Co-Innovation Center for Modern Production Technology of Grain Crops, Yangzhou University, Yangzhou, P. R. China

## Abstract

High temperature (temperature over 35 °C) is an extremely important environmental factor that affects the maize grain quality in Southern China. The effects of heat stress after pollination on grain protein and starch deposition and activities of involved enzymes were studied in a pot trail in 2014 and 2015. Results showed that grain dry weight reductions at maturity were 19.8% and 19.1%, whereas starch contents (mg g^−1^) were reduced by 3.0% and 3.3%, and starch accumulation (mg grain^−1^) were reduced 22.2% and 21.8% in 2014 and 2015, respectively. Protein content was decreased by heat stress before 15 DAP and increased thereafter. At maturity, protein contents (mg g^−1^) were increased by 24.5% and 25.3% in 2014 and 2015, while protein accumulation (mg grain^−1^) were not affected by heat stress. In response to heat stress, glutamate synthase activity was enhanced by 29.1–82.9% in 2014 and 2.0–141.8% in 2015, whereas glutamine synthetase activity was reduced by 1.9–43.5% in 2014 and 0.1–27.4% in 2015 throughout the grain filling. The activities of sucrose phosphate synthase were decreased by heat stress at 10–25DAP (12.7–32.0%) in 2014 and 15–20 DAP (23.2–27.5%) in 2015, and activities of sucrose synthase were decreased by heat stress at 5–15 DAP (20.0–45.0%) in 2014 and 15 DAP (22.0%) in 2015, repectively. The activities of enyzmes that involved in starch synthessis were all suppressed by heat stress during grain filling, and the reduction of adenosine diphosphate-glucose pyrophosphorylase, soluble starch synthase, and starch branching enzyme were decreased by 21.3–43.1%, 19.1–29.2%, and 7.0–45.6% in 2014 and 1.8–78.5%, 21.4–51.2%, and 11.0–48.0% in 2015, respectively. Conclusively, grain weight and starch deposition were suppressed by heat stress due to the decreased activities of enzymes involved in starch synthesis, and the increased protein content was due to the enhanced activity of glutamate synthase.

## Introduction

Starch is the main component of maize grain, followed by protein, which determines the grain quality. The grain quality is affected by the interaction of genotype and environmental factors, which change grain starch and protein formation^1,2^. Among the environmental factors, high temperature (over 35 °C), especially during summer in Southern China, is the most important factor that affects maize grain quality. Wang and Frei^3^ found that heat stress during grain filling reduces the cereal starch content, and increases the protein content. The change in grain components and contents was due to changes of enzymes involved in starch and protein synthesis. Duke and Doehlert^4^ observed that the activities of adenosine diphosphate (ADP)-glucose pyrophosphorylase (AGPase), aldolase, acid invertase, and acid phosphatase in normal maize grain were decreased by heat stress during grain filling. Heat stress during grain filling decreased the activities of enzymes involved in starch metabolism in normal maize, such as sucrose synthase (SuSy), AGPase, glucokinase, soluble starch synthase (SSS) and starch branching enzyme (SBE), which restricted the accumulation of starch^5,6^. The normal maize grain protein accumulation was decreased, whereas the concentration of protein components, such as glutelin, albumin, and globulin, was relatively increased by 4 day heat stress at 5 days after pollination (DAP)^7^. Heat stress during grain filling decreased the activities of sucrose phosphate synthase (SPS) and SuSy, which inevitably led to decreased sucrose content in sweet maize^8^.

In rice, heat stress decreased the activities of glutamine synthetase (GS), but increased the activities of glutamate synthase (GOGAT) and glutamate pyruvate transaminase, resulting in the increase of protein and amino acid contents^9^. The activities of SuSy and SBE in rice were increased by heat stress at the early grain development stage but decreased thereafter^10^. Moreover, SSS activity was decreased by heat stress during grain filling^11^. Heat stress strongly suppressed the expression of the genes related to sucrose and starch synthesis-related enzymes, producing chalky grains due to the reduced deposition of starch^12,13^. The high protein content was attributed to the decreased transcripts of the glutelin and/or prolamin family genes in the middle and late filling stages^14^. In wheat, SuSy in grain was enhanced before 14 DAP and reduced thereafter^15^. The low starch under heat stress may be due to the decreased activities of SSS^16^ and AGPase^17^.

In our previous study, the waxy maize grain starch content was decreased, whereas protein content was increased, resulting in the change of grain quality^18,19^. However, in-depth understanding of the activities of enzymes involved in the synthesis of starch and protein is still lacking. Therefore, in the present study, the activities of enzymes involved in starch and protein metabolism in response to heat stress during grain filling were clarified to help improve our understanding of the mechanism of change in grain components under high temperature conditions.

## Results

### Grain weight accumulation

**Figure 1:**
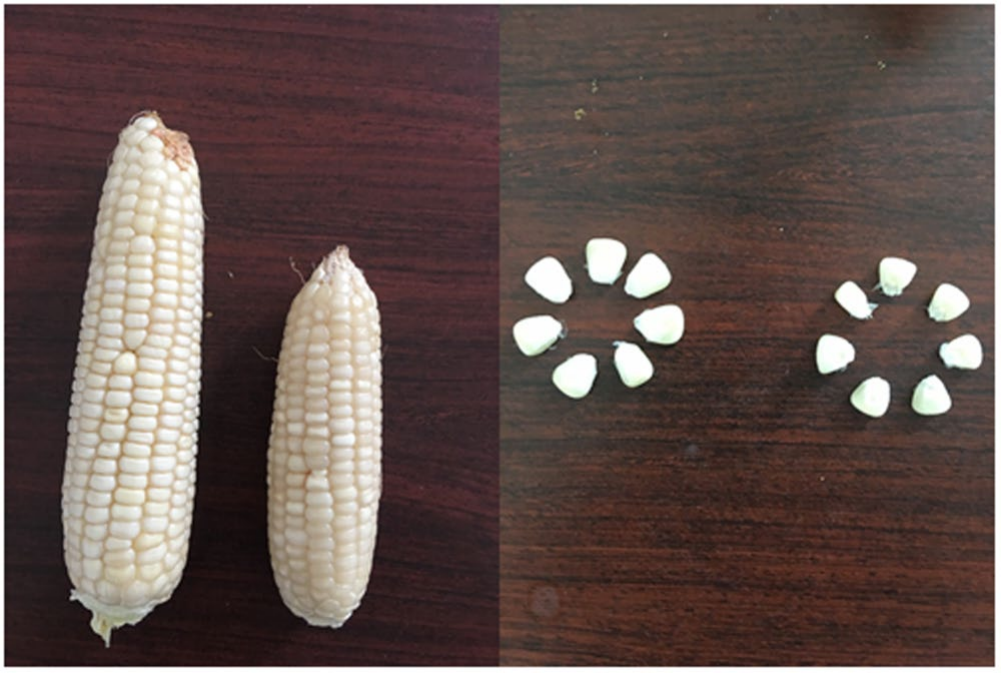
Pictures of ear and grains under ambient (left) and high-temperature (right) conditions.

**Figure 2:**
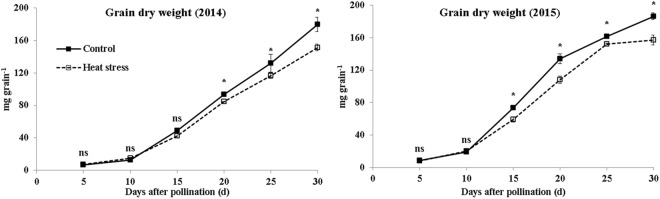
Grain dry weight during grain filling under ambient and high-temperature conditions. Bars denote standard errors from three replicates, and one-way ANOVA was used to test for significance at *p* < 0.05 level.

The size of ear and grain was significantly decreased by heat stress during grain filling (Fig. 1). The grain weight increased gradually with the development of grain filling, but the increase became sharply after 10 DAP (Fig. 2). No difference was observed between the ambient and high temperature at 5–10 DAP in 2014 and 2015, whereas the grain weight was suppressed by heat stress with grain development. The reductions of grain dry weight at maturity were 19.8% and 19.1% in 2014 and 2015, respectively (Table 1).

**Table 1:** The grain weight and contents of starch and protein at maturity under ambient and high-temperature conditions in two years.

Year	Temperature	Grain dry weight (mg grain^−1^)	Starch content (mg g^−1^)	Starch accumulation (mg grain^−1^)	Protein content (mg g^−1^)	Protein accumulation (mg grain^−1^)
2014	Control	222.2 a	627.0 b	139.3 a	82.5 b	18.3 a
	Heat stress	178.3 c	608.2 c	108.4 c	94.7 a	16.9 b
2015	Control	207.1 b	633.0 a	131.1 b	77.6 c	16.1 b
	Heat stress	167.4 d	612.4 c	102.5 c	97.2 b	16.3 b

Values are means of three pots (three replicates). Values followed by different lowercases in the same column indicate significant differences among treatments at *P* < 0.05 level (*LSD* test).

### Starch deposition

**Figure 3:**
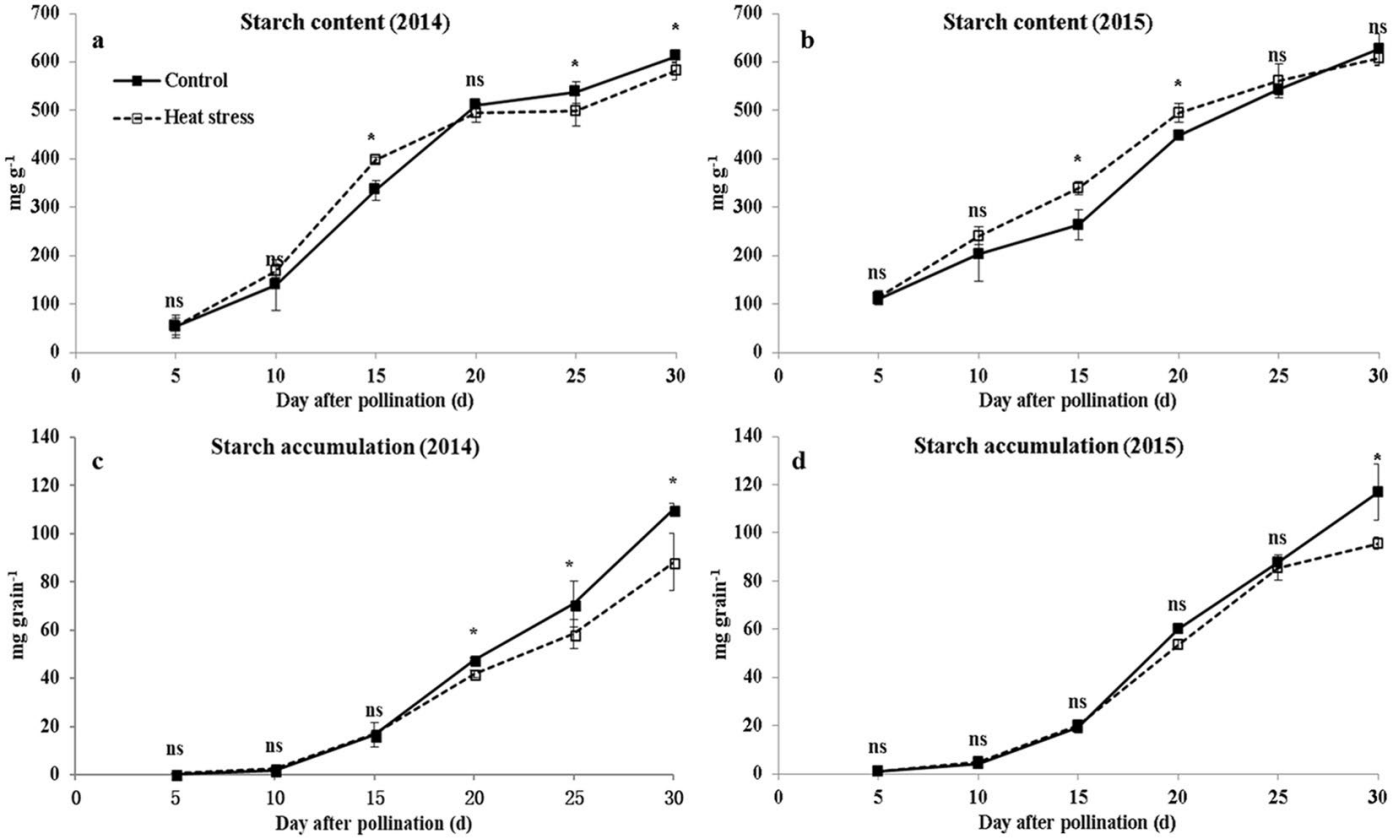
Grain starch accumulation during grain filling under ambient and high-temperature conditions. (**a**,**c** and **b**,**d**) are the grain starch content and starch accumulation in 2014 and 2015, respectively. Bars denote standard errors from three replicates, and one-way ANOVA was used to test for significance at *p* < 0.05 level.

The grain starch content was not affected by heat stress at 5–10 DAP but was increased by heat stress at 15 DAP (18.8%) in 2014 and at 15 (29.0%) and (10.6%) 20 DAP in 2015. Thereafter, the value in 2015 was not affected by heat stress, while it was decreased by 4.8–7.0% in 2014, respectively (Fig. 3a,b). At maturity, the starch contents were decreased by 3.0% and 3.3% in 2014 and 2015, respectively (Table 1). The starch deposited at 5–15 DAP was similar under ambient and high temperature for both years. The starch deposition was suppressed by heat stress at 15 DAP in 2014 but was significantly decreased at maturity (up to 22.2%). In 2015, the starch deposition was decreased only after 30 DAP, which were 18.2% and 21.8% lower than that under ambient temperature at 30 DAP and maturity, respectively (Fig. 3c,d; Table 1).

### Protein accumulation

**Figure 4:**
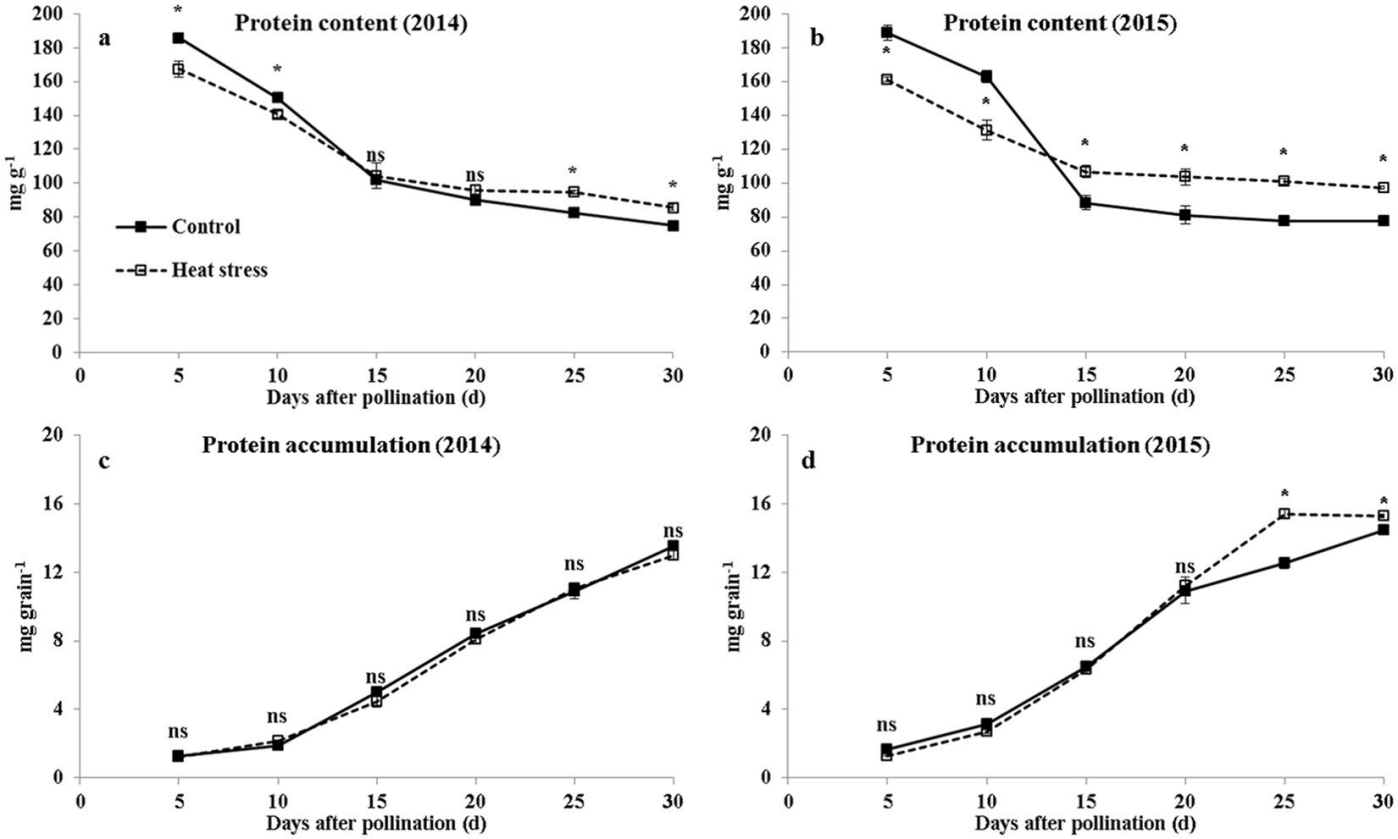
Grain protein accumulation during grain filling under ambient and high-temperature conditions. (**a**,**c** and **b**,**d**) are the grain protein content and protein accumulation in 2014 and 2015, respectively. Bars denote standard errors from three replicates, and one-way ANOVA was used to test for significance at *p* < 0.05 level.

Compared with that in ambient temperature, the grain protein content under heat stress was lower before 10 DAP (6.6–9.9% in 2014 and 14.6–19.4% in 2015). At 15–30 DAP, the grain protein content was increased by heat stress (2.1–14.8% in 2014 and 20.9–30.1% in 2015) (Fig. 4a,b). At maturity, the protein contents were increased by 24.5% and 25.3% in 2014 and 2015, respectively (Table 1). The protein accumulation in 2014 was not affected by heat stress throughout the grain filling, while it was increased by 22.6% at 25 DAP and 5.6% at 30 DAP but not affected at maturity in 2015 (Fig. 4c,d; Table 1).

### Activities of enzymes involved in protein synthesis

**Figure 5:**
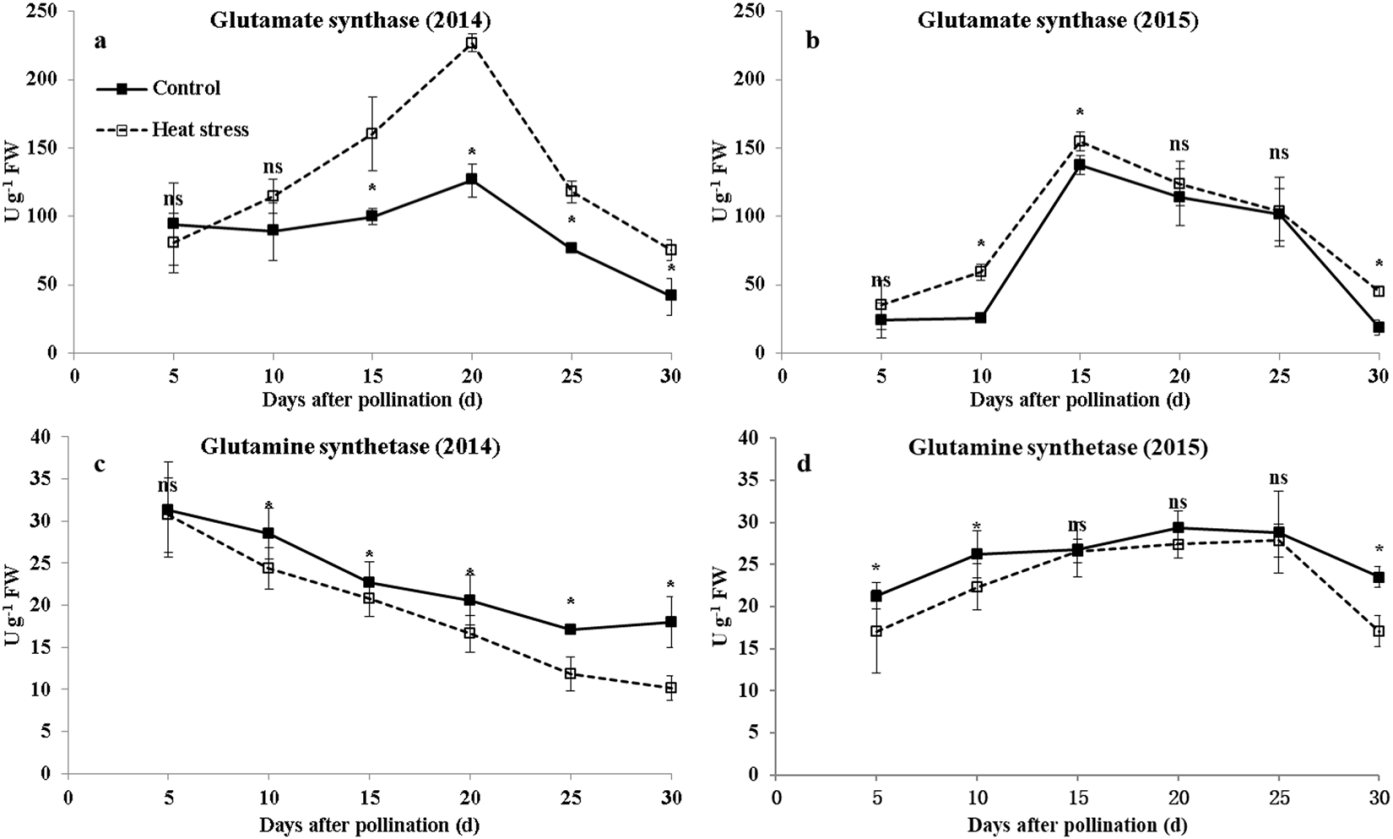
Activities of GOGAT and GS during grain filling under ambient and high-temperature conditions. (**a**,**c** and **b**,**d**) are the activities of GOGAT and GS in 2014 and 2015, respectively. Bars denote standard errors from three replicates, and one-way ANOVA was used to test for significance at *p* < 0.05 level.

GS and GOGAT are two key enzymes involved in protein synthesis. The activities of GOGAT in grains were not affected by heat stress at 5 DAP. However after 10 DAP, they were increased by heat stress (29.1–82.9% in 2014 and 2.0–141.8% in 2015) (Fig. 5a,b). The activities of GS were decreased by heat stress throughout the grain filling (1.9–43.5% in 2014 and 0.1–27.4% in 2015) (Fig. 5c,d).

### Activities of enzymes involved in starch synthesis

**Figure 6:**
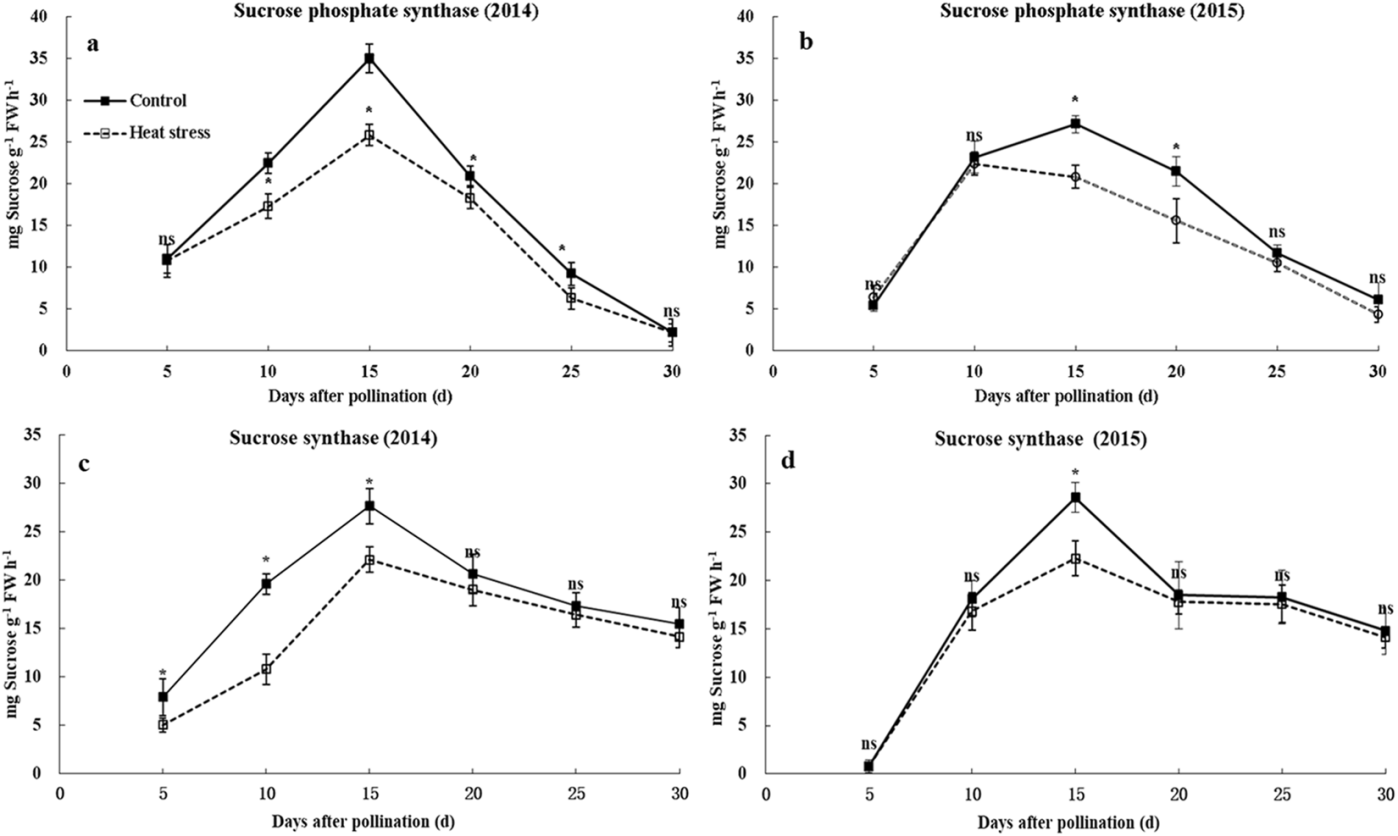
Activities of SPS and SuSy during grain filling under ambient and high-temperature conditions. (**a**,**c** and **b**,**d**) indicate the activities of SPS, SuSy in 2014 and 2015, respectively. Bars denote standard errors from three replicates, and one-way ANOVA was used to test for significance at *p* < 0.05 level.

Sucrose is the main transport substrate for starch formation in cereals. The activities of SPS were decreased by heat stress at 10–25DAP (12.7–32.0%) in 2014 and 15–20 DAP (23.2–27.5%) in 2015 (Fig. 6a,b). The activities of SuSy were decreased by heat stress at 5–15 DAP (20.0–45.0%) in 2014 and 15 DAP (22.0%) in 2015, which was similar between ambient and high temperature conditions in other stages (Fig. 6c,d).

**Figure 7:**
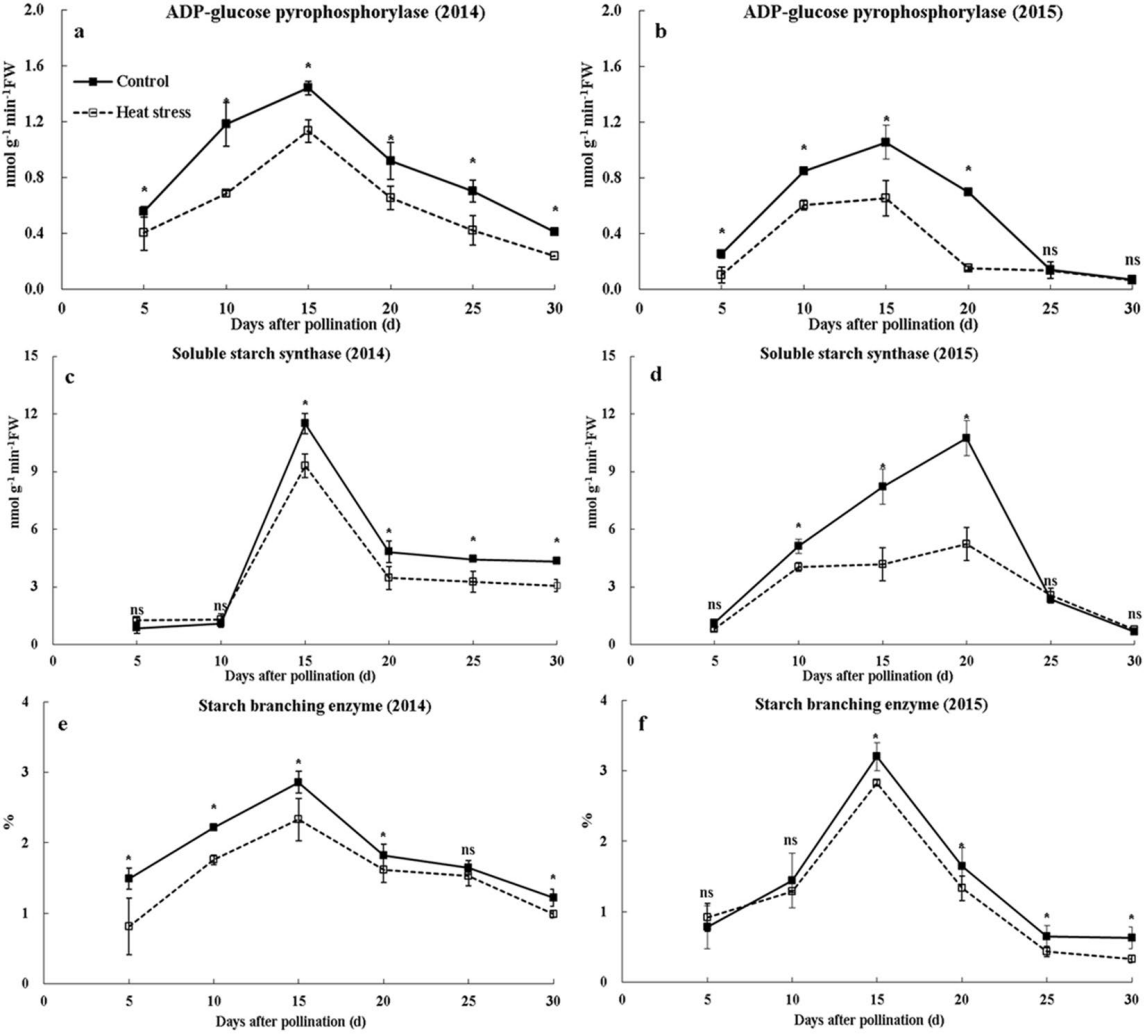
Activities of AGPase, SSS, and SBE during grain filling under ambient and high-temperature conditions. (**a**,**c**,**e** and **b**,**d**,**f**) indicate the activities of AGPase, SSS, and SBE in 2014 and 2015, respectively. Bars denote standard errors from three replicates, and one-way ANOVA was used to test for significance at *p* < 0.05 level.

Grain starch synthesis was regulated by AGPase, SSS and SBE. In the present study, the activities of AGPase, SSS, and SBE initially increased and then decreased afterward with grain development, and these parameters were reduced by heat stress (Fig. 7). The activities of AGPase, SSS, and SBE were decreased by 21.3–43.1%, 19.1–29.2%, and 7.0–45.6% in 2014 and 1.8–78.5%, 21.4–51.2%, and 11.0–48.0% in 2015, respectively.

## Discussion

Zhang *et al*.^20^ reported that the grain filling rate of normal maize under heat stress condition was accelerated at the early stage but restrained thereafter, and that grain weight was not affected before 20 DAP and was suppressed with grain development. In the present study, the grain weight increment was similar under ambient and high temperature conditions at 5–10 DAP and was restrained afterward by heat stress. The grain dry weight at maturity was decreased by 19.8% and 19.1% in 2014 and 2015, as reported early^21^. However, in our previous study, the grain weight was not affected by heat stress before 17 DAP, increased at 15–30 DAP and suppressed thereafter^18^. The discrepancy may be due to the difference in ambient temperature (27.4 °C/15.6 °C) after pollination, which was lower than that in the present study (29.0 °C/22.0 °C in 2014 and 28.2 °C/21.5 °C in 2015).

Many studies reported that heat stress during grain filling increases the protein content and reduces the starch content in cereal grains^3,22,23^. In the present study, the grain starch content was increased first and decreased thereafter, similar to our previously finding^18^. The grain starch content at maturity was decreased 3.0% and 3.3% in 2014 and 2015. Zhang *et al*.^20^ also observed that the starch content in normal maize grain at 28 and 40 DAP was decreased by heat stress at 17–28 DAP. The grain starch deposition was suppressed by heat stress after 20 DAP, and the reduction (22.0%) at maturity was mainly due to the decrease of grain weight (19.5%), and low starch content (3.1%, average of 2014 and 2015). However, research on rice found that the starch deposition was only suppressed in a heat-susceptible variety, but was increased initially and then became unaffected by heat stress in a heat-tolerant variety^11^. Based on this finding, breeding a heat-tolerant waxy maize variety may be suitable for production in high temperature climates. The grain protein content was decreased by heat stress before 15 DAP and increased thereafter. Under high temperature condition, the grain protein content at maturity was increased by 24.5% in 2014 and 25.3% in 2015, while grain protein deposition was not affected in both years, indicated the increase of protein content was only a concentration effect. This is due to adverse environmental factors that promote post-flowering leaf senescence tend to favor grain protein accumulation over starch deposition, because the production and translocation of carbohydrates to the grain is more sensitive to environmental stresses than protein accumulation^3^.

The assimilation of inorganic nitrogen for incorporation into organic compounds such as proteins and nucleic acids is coupled with the formation of glutamate by GOGAT as part of the GS/GOGAT cycle^24^. Increased activities of GS and GOGAT can enhance the nitrogen metabolism and accelerate the protein synthesis and conversion of amino acids^9,25^. In the present study, GOGAT activities were increased, whereas GS activities were decreased by heat stress. Similar results were observed in rice^9^ and wheat^15^. The low activities of GS in grain under heat stress did not restrain protein synthesis, which may due to the glutamine was mainly dependent on the GS catalytic synthesized the glutamine in leaf and transported to grain^9^. The reduction of GS activity in grains under heat stress did not affect protein accumulation. Thus, GS may not be the key enzyme that influences protein synthesis.

Sucrose is the main transport substrate for starch formation in cereals, and its synthesis is mainly regulated by SPS and SuSy^26,27^. SPS is the primary regulator for controlling biosynthesis and accumulation of sucrose and plays an important role in the translocation and distribution of photoassimilates in higher plants^28^. SuSy is a key enzyme in plant carbohydrate metabolism and utilizes the conserved energy of the transport metabolite sucrose for the formation of nucleotide sugars as precursors for starch and cellulose biosynthesis^29,30^. The activities of SPS were decreased by heat stress at 10–25 DAP and 15–20 DAP in 2014 and 2015, respectively. The activities of SuSy were decreased by heat stress before 15 DAP. Later on, this difference no longer existed. A study on sweet maize showed that the activities of SuSy were decreased throughout grain filling^8^. The reduced activities of SPS and SuSy restrained sucrose synthesis^26,30^ and increased grain starch content before 15 DAP. This is because SuSy activity regulates sucrose synthesis, sustains sucrose unloading and entering in metabolism, and maintains cell division or starch accumulation^31^. Studies in rice^10^ and wheat^15^ found that the activities of SuSy were increased initially by heat stress and reduced thereafter. However, the activities of SuSy were only decreased by heat stress for heat-susceptible rice varieties^11^, indicating the importance of breeding heat-tolerant varieties for waxy maize production.

Grain starch synthesis was mainly regulated by AGPase, SSS, and SBE, the activities of which were reduced by heat stress. Similar results were reported in normal maize^5,6,20^, wheat^15–17^, and rice^11,32^. However, the starch content before 20 DAP was increased, and starch accumulation per grain was not affected by heat stress, which was inconsistent with the decreased activities of AGPase, SSS, and SBE. This is probably because AGPase activity was not directly correlated with the starch synthesis rate, and increased SuSy activity favored starch accumulation^29–31^. The change in AGPase and SSS activities at 25–30 DAP was consistent with the starch content in 2014 and 2015. Some studies in rice and wheat^10,33–36^ observed that the activities of AGPase, SSS, and SBE were increased initially by heat stress and then decreased, and that the expression levels of genes that control these enzymes changed correspondingly. Therefore, the effects of heat stress on the expression of key genes that control starch synthesis need to be elaborated in future studies. In addition, using anti-sense technology to inhibit key enzyme activity could help us to understand its role in starch synthesis. As hydrolytic enzymes such as α-amylase are involved in starch synthesis and degradation, determining their function on starch morphology and quality could increase our understanding on waxy maize grain utilization^12,13,37,38^.

## Materials and Methods

### Plant materials and growth conditions

Suyunuo5, a well-known waxy maize variety covering large plantation areas in Southern China, was analyzed in farm at Yangzhou University (Yangzhou, China) in 2014 and 2015. Seeds were sown in March 15 and transplanted to plastic pots (two plants per pot, with one plant retained at the jointing stage) in March 28. Each plastic pot was 38 cm in height and 43 cm in diameter. It was loaded with 30 kg of sieved sandy loam soil. The plants were provided a basal dressing of 10 g per pot (commercial fertilizer, N/P_2_O_5_/K_2_O = 15%/15%/15%) at transplantation and a top dressing of 6.6 g per pot (commercial urea, N = 46%) at the jointing stage.

After artificial pollination, the plants were moved to a greenhouse for heat stress treatment. The temperature of the greenhouse was 35.0 °C in the day (06:00–18:00). From 18:00 of the previous day to 06:00 of the next day, the door and windows of the greenhouse were left open to ensure that the indoor temperature at night was identical to the outdoor temperature. Outdoor conditions with temperatures (day/night) of 29.0 °C/22.0 °C (2014) and 28.2 °C/21.5 °C (2015) were taken as the control^21^.

### Samples preparation

Three ears were harvested at 5, 10, 15, 20, 25, 30 DAP and maturity. After photoed the ear and grains, the grain numbers were counted, some of the grains (approximately 50–60 grains at middle position) were immediately frozen in liquid N_2_ after being stripped from the ears and then stored at −75 °C until analysis. 100-grains randomly selected from the left grains on independent ear were deactivated at 105 °C for half an hour and dried to consistent weight at 60 °C. Then, the grain weight was determined based on three independent ears as three replicates. After drying, the grains were ground and passed through a 100-mesh filter (d = 0.149 mm) for grain starch and protein content analysis.

### Protein and starch analysis

The grain starch content was determined using the anthrone-sulfuric acid method^39^. The nitrogen content was determined using the Kjeldahl method^40^, and protein content was determined using the following formula: protein content = nitrogen content × 6.25. Protein (starch) accumulation was determined using the following formula: grain dry weight × protein (starch) content.

### Assays of SPS and SuSy activities

All procedures for enzyme extraction were carried out in ice (0 °C–2 °C). The embryo and pericarp of the grains were removed, and approximately 1 g fresh weight of the lyophilized samples were homogenized in the extraction buffer containing 50 mM HEPES-NaOH (pH 7.5) followed by centrifugation at 10,000 × *g* for 10 min^8^. The supernatant was used for enzyme assays.

### SuSy (EC 2.4.1.13)

SuSy assay followed the method proposed earlier^8^ with modifications. Fifty microliters of enzyme solution was added with 50 μL of HEPES-NaOH (pH 7.5), 20 μL of 100 mM UDPG, 20 μL of 100 mM fructose and 20 μL of 50 mM MgCl_2_ and reacted at 30 °C for 30 min. The reaction was terminated by adding 0.2 mL of 2 M NaOH, and the solution was heated for 10 min in boiling water. Then, 2.0 mL of 30% HCl was added, and the solution was kept at 80 °C for 10 min. Subsequently, 1 mL of 1% resorcinol was added and kept for another 10 min. After the solution was cooled to room temperature, it was mixed with 3.64 mL of deionized water. The optical density (OD) value was read at 480 nm. The unit of the activity was defined as the amount of sucrose produced.

### SPS (EC 2.4.1.14) activity

The procedure for SPS determination was identical to that of SuSy except 20 μL of 100 mM fructose was replaced with 20 μL of 100 mM fructose-6-phosphate^8^.

### Assay of GS and GOGAT activities

The assays of GS and GOGAT followed the method proposed earlier^9^. Simply, the embryo and pericarp-removed grains (1 g fresh weight) were homogenized with 5 mL of buffer solution (pH 7.5, 100 mM Tris-NaOH, 6 mM MgCl_2_, 2 mM EDTA, and 40 mM 2-mercaptoethanol) precooled in ice, followed by centrifugation at 10,000 × *g* for 20 min. The supernatant was used for enzyme assays.

### GS (EC 6.3.1.2) activity

Twenty microliters of enzyme solution was added into 0.1 mL of buffer solution (pH 7.0, 0.25 mM imidazole-HCl, 0.3 mM monosodium glutamate, 30 mM ATP-Na and 0.5 mM MgSO_4_ and 0.02 mL of 100 mM hydroxylamine. The reaction was conducted at 35 °C for 15 min and terminated by adding 0.1 mL FeCl_3_. The reaction solution without ATP and glutamate was set as the control. After centrifugation at 8,000 × *g* for 5 min, the OD value was read at 540 nm. The unit of GS activity was defined as the amount of enzyme that catalyzes the production of 1 μmol of glutamyl-hydroxamate per minute.

### GOGAT (EC 1.4.1.13) activity

One hundred microliters of enzyme solution was added into 1 mL of reaction solution (pH 7.5, 100 mM potassium phosphate, 2 mM α-oxoglutarate, 0.2 mM NaOH, 10 mM L-glutamine). The reaction was conducted at 30 °C for 30 min and stopped by boiling in water for 30 s. The OD value was read at 340 nm immediately. The reaction solution without L-glutamine was used as control. One unit of GOGAT activity was defined as the amount of enzyme which consumed 1 μmol of NADP per minute.

### Assay of enzymes involved in starch synthesis

The embryo and pericarp-removed grains (1 g fresh weight) were homogenized with 5 mL of buffer solution (pH 7.5, 100 mM of HEPES-NaOH, 8 mM of MgCl_2_, 2 mM of EDTA, 50 mM of 2-mercaptoethanol, 12.5% (v/v) glycerol and 5% (w/v) insoluble polyvinylpyrrolidone-40). The homogenate was centrifuged at 10,000 × *g* for 5 min, and the resulting supernatant was used for enzyme preparation, unless otherwise stated. The assays of the AGPase (EC 2.7.7.27), SSS (EC 2.4.1.21) and SBE (EC 2.4.1.18) followed the method proposed by Nakamura *et al*.^41^ with slight modifications.

AGPase – The assay was conducted in 100 mM HEPES-NaOH (pH 7.5), 1.2 mM ADPG, 3 mM PPi, 5 mM MgCl_2_, 4 mM DTT, and enzyme preparation (100 μL) in a reaction mixture of 650 μL. After 20 min at 30 °C, the reaction was terminated by heating the mixture in boiling water for 30 s. The resulting solution was transferred to an Eppendorf tube and centrifuged at 10,000 × *g* for 10 min. A portion (500 μL) of the supernatant was taken and mixed with 15 μL of 10 mM NADP. The enzymic activity (nM g^−1^ min^−1^ FW) was assayed by measuring the increase in absorbance at 340 nm after the addition of 1 μL each of P-glucomutase (0.4 unit) and G6P dehydrogenase (0.35 unit).

SSS – The assay was conducted in 50 mM HEPES-NaOH (pH 7.5), 1.6 mM ADPG, 0.7 mg amylopectin, 15 mM DTT, and enzyme preparation (100 μL) in a reaction mixture of 280 μL. Twenty minutes after the start of the reaction at 30 °C, the enzyme was deactivated by placing the mixture in a boiling water bath for 30 s. Then, the mixture was added with 100 μL of a solution containing 50 mM HEPES-NaOH (pH 7.5), 4 mM PEP, 200 mM KCl, 10 mM MgCl_2_, and pyruvate kinase (1.2 unit) and incubated for 30 min at 30 °C. The ADP produced by the starch synthase reaction was converted to ATP, and the resulting solution was heated in a boiling water bath for 30 s. Then, it was subjected to centrifugation at 10,000 × *g* for 5 min. The supernatant (300 μL) was mixed with a solution of 50 mM HEPES-NaOH (pH 7.5), 10 mM glucose, 20 mM MgCl_2_, and 2 mM NADP. The enzyme activity (nM g^−1^ min^−1^ FW) was measured as the increase in absorbance at 340 nm after the addition of 1 μL each of hexokinase (1.4 unit) and G6P dehydrogenase (0.35 unit).

SBE – The assay was conducted in 50 mM HEPES-NaOH (pH 7.5), 5 mM G1P, 1.25 mM AMP, phosphorylase a (54 unit), and enzyme preparation in a reaction mixture of 200 μL. The reaction was terminated by the addition of 50 μL of 1 N HC1. The solution was mixed with 500 μL of dimethylsulfoxide and added with 700 μL of 0.1% I_2_ and 1% KI. The enzymic activity was assayed spectrophotometrically at 540 nm. One unit of enzyme activity (%) was defined as the amount causing an increase in absorbance of 1 U at 540 nm in 1 min. ΔOD540 nm = (OD540 0-min-OD540 t-min) × 100%/OD540 0-min (t > 0).

### Statistical analysis

The data reported in all figures are expressed as averages of three independent ears. Data were subjected to ANOVA with the least significant difference (LSD) test at 0.05 probability level by using the Data Processing System (version 7.05).
